# Stabilization of Graphene Oxide Dispersion in Plasma-like Isotonic Solution Containing Aggregating Concentrations of Bivalent Cations

**DOI:** 10.3390/pharmaceutics15102495

**Published:** 2023-10-19

**Authors:** Marcin Z. Krasoń, Anna Paradowska, Martyna Fronczek, Mateusz Lejawa, Natalia Kamieńska, Michał Krejca, Anna Kolanowska, Sławomir Boncel, Marek W. Radomski

**Affiliations:** 1 Silesian Park of Medical Technology Kardio-Med Silesia, 41-800 Zabrze, Poland; anparadowska@wp.pl (A.P.); m.fronczek@kmptm.pl (M.F.); mateusz.lejawa@gmail.com (M.L.); 2 Cardiac Surgery Department, Medical University of Łódź, 90-419 Łódź, Poland; mkrejca@wp.pl; 3 Department of Cardiac, Vascular and Endovascular Surgery and Transplantology, Medical University of Silesia in Katowice, Silesian Center for Heart Diseases in Zabrze, 41-800 Zabrze, Poland; n_rosz@poczta.onet.pl; 4 Department of Pharmacology, Faculty of Medical Sciences in Zabrze, Medical University of Silesia in Katowice, 40-055 Katowice, Poland; 5 Department of Organic Chemistry, Bioorganic Chemistry and Biotechnology, Faculty of Chemistry, Silesian University of Technology, 44-100 Gliwice, Poland; anna.kolanowska@polsl.pl (A.K.); slawomir.boncel@polsl.pl (S.B.); 6 Centre for Organic and Nanohybrid Electronics, Silesian University of Technology, 44-100 Gliwice, Poland; 7 Department of Anatomy, Physiology and Pharmacology, College of Medicine, University of Saskatchewan, Saskatoon, SK S7N 5E5, Canada; marek.radomski@usask.ca

**Keywords:** graphene oxide, aqueous dispersion, stability of dispersion, nanoparticle size, ion-induced aggregation, bovine serum albumin

## Abstract

Graphene oxide’s (GO) intravascular applications and biocompatibility are not fully explored yet, although it has been proposed as an anticancer drug transporter, antibacterial factor or component of wearable devices. Bivalent cations and the number of particles’ atom layers, as well as their structural oxygen content and pH of the dispersion, all affect the GO size, shape, dispersibility and biological effects. Bovine serum albumin (BSA), an important blood plasma protein, is expected to improve GO dispersion stability in physiological concentrations of the precipitating calcium and magnesium cations to enable effective and safe tissue perfusion. Methods: Four types of GO commercially available aqueous dispersions (with different particle structures) were diluted, sonicated and studied in the presence of BSA and physiological cation concentrations. Nanoparticle populations sizes, electrical conductivity, zeta potential (Zetasizer NanoZS), structure (TEM and CryoTEM), functional groups content (micro titration) and dispersion pH were analyzed in consecutive preparation stages. Results: BSA effectively prevented the aggregation of GO in precipitating concentrations of physiological bivalent cations. The final polydispersity indexes were reduced from 0.66–0.91 to 0.36–0.43. The GO-containing isotonic dispersions were stable with the following Z-ave results: GO1 421.1 nm, GO2 382.6 nm, GO3 440.2 nm and GO4 490.1 nm. The GO behavior was structure-dependent. Conclusion: BSA effectively stabilized four types of GO dispersions in an isotonic dispersion containing aggregating bivalent physiological cations.

## 1. Introduction

Graphene is a material with unique features attractive for both industrial and medical applications. Each layer of graphene has a two-dimensional structure of a single-atom thickness. Carbon atoms have an sp^2^-hybridization, packed in a honeycomb lattice. This nanomaterial is 200 times stronger than steel, with a Young’s modulus of 1.02 TPa (for steel: 210 MPa) and Poisson’s ratio 0.149 (for steel: 0.30) [1]. Also, thermal (up to 5.30 × 10^3^ W/(m·K) [2] and electrical (7200 S/m) [3] conductivities of graphene are remarkably high. Being a zero-band gap semiconductor in its single-layered form, [1] graphene (and also graphenoids, i.e., graphene of various layers) form several types of chemical bonds (van der Waals, hydrophobic, electrostatic, π-π stacking, covalent bonds, etc.), and can be obtained by a reduction of the synthesized via graphite oxidation graphene oxide (GO) toward more individualized forms up to the industrial scale. 

GO is an important widely used ‘variant of graphene’ that is hydrophilic [4] and biocompatible [5], presenting an increased dispersibility in water. The added chemical groups in GO change the particle surface and alter the conductivity of GO dispersions. Oxygen-containing epoxy, hydroxyl and carboxylic groups (prone to creating the subsequent chemical bonds and changing carbon hybridization) can be positioned both on the basal plane and the edges of the 2D nanoparticles. Khoei et al. [6], applying molecular dynamics (MD), reported the bending and/or formation of wrinkles in the basal plane of the GO nanoparticles upon the introduction of higher concentrations of hydroxyl (OH-), epoxy (C,C,>O), keto (O=C<) and carboxylic (-COOH) groups. The observed structural changes were also dependent on the type and location of (C,C,>O) and -OH oxygen moieties. The addition of these functionalities made GO more ductile, displaying a lower Young modulus and lower maximal tensile stress than the pristine graphene [6]. The presence of oxygen-containing chemical moieties together with a wrinkled, rough surface structure improves the GO wettability through mutually enhancing the chemical and surface interactions [7]. It is crucial for increasing the intravascular applicability of GO under physiological regimes, where this substance is rarely used due to the fact that high concentrations of tissue sodium (>30 mM), magnesium (>0.7 mM) and calcium (>0.4 mM) ions destabilize aqueous dispersions via the aggregation of GO (nano)particles [8]. This situation emerges as the most possible reason for why reports on intravascular, circulatory and ex vivo experimental GO applications are scarce and rarely accompanied with precise characteristics of GO nanoparticles in-tissue stabilization strategies. We focus on an improved GO water dispersibility and underestimate the precipitating cations in tissue and blood milieu. Organ perfusion solutions and blood plasma themselves contain precipitating concentrations of at least two bivalent cations (Mg, Ca) able to create stable bonds between the oxygen-containing groups of neighboring GO layers. The problem of the aggregation of GO in tissues is not yet fully explored and is important, especially when GOs are being studied in numerous biomedical applications such as advanced 3D tissue scaffolds for neurons, adipose stem cells, bone cells, human osteoblasts and myoblasts [9], in fluorescent and electrochemical biosensors tested for early cancer diagnostics [10], intravascular anticancer drug delivery systems [11,12], wound healing [13,14] and bone regeneration [15]. GO was also reported to demonstrate antibacterial [16] and infection-preventive properties [17].

The biological activity of GO (nano)particles is affected by their interactions with plasma proteins. These interactions depend on the surface of the particle [18], and can be personalized by the individual serum protein patterns, which may be different in specific diseases [19]. The formation of such protein corona can alter the nanoparticle biological functions and organ effects. Indeed, the toxic effects of GO on human lung cell carcinoma cells are attenuated in the presence of 10% fetal bovine serum [20] or bovine serum albumin (BSA) [21]. The modification of the enzymatic proteins by GO can also change their biochemical functions [22]. 

The aim of this study was to report an originally developed, BSA-based procedure for the effective stabilization of four different commercially available GO samples of various morphology and a variable oxygen content in the highly demanding perfusion solution containing precipitating amounts of magnesium, calcium and sodium cations for biological use. Our main interest was to obtain the maximal increase in the monodispersity of the GO size and elimination of agglomerates to prepare dispersions (containing GO particles of the unified particle size) that could be effective and fully predictable in the nanoparticle final organ effects and microvascular organ perfusion. 

## 2. Materials and Methods

### 2.1. Analyzed Materials

Four different GO water dispersions were tested: GO1: few-layered GO containing <49% oxygen (O) concentration 5 mg/mL in water dispersion (Nano Carbon, Warsaw, Poland, company liquidated) (few layer graphene oxide; form: stable paste or dispersion; production method: oxidation of powdered graphite by modified Hummers method; Raman spectroscopy result: ID/IG >1,87; elemental analysis–percentage by weight: C: >45%, H: <2.5%, N: <0.5%, O: <49%, others < 4%; XRF impurities analysis: S(2.5%) > Ca(1%) > Mn(0.5%) > K(0.3%) > Cl(0.08%) = Fe(0.08%) > Cu(0.02%) = Zn(0.02%) > Ni(0.007%) > Cr(0.006%); color: dark brown; GO2: predominantly single-layered GO with 41–50% of O; concentration: 4 mg/mL in water dispersion; form: dispersion of graphene oxide sheets; particle size: D90 29.05–32.9 μm, D50 14.30–16.6 µm, D10 5.90–6.63 µm; color: yellow-brown; odor: odorless; dispersibility: polar solvents; solvent: water; pH: 2.2–2.5; monolayer content (measured in 0.5 mg/mL) >95% (4 mg/mL concentration tends to agglomerate the GO flakes, and dilution followed by slight sonication is required in order to obtain a higher percentage of monolayer flakes); elemental analysis (sample preparation: 2 g of 4 wt% GO in water was dried under vacuum at 60 °C overnight): C: 49–56%, H: 0–1%, N: 0–1%, O: 41–50%, S: 2–4% (Graphenea, San Sebastian, Spain https://www.graphenea.com (accessed on 12 October 2023); GO3: 15–20-layered GO, 4–10% edge-oxidized, containing up to 11% of O, concentration of 1 mg/mL, water dispersion (Sigma-Aldrich, Poznań, Poland, Product of Garmor Inc., Orlando, Fl, USA), (graphene oxide 4–10% edge-oxidized; form: suspension; bulk density: ~1.8 g/cm^3^; number of layers: 15–20; appearance (color): very dark brown to black; carbon (dry basis): ≥50%; oxygen (dry basis): ≤11%; residue on evaporation: 0.9–1.4 mg/mL); and GO4: *N*-doped GO, ammonium-functionalized, concentration 1 mg/mL, water dispersion (Sigma-Aldrich, Poland, cat. No. 791520) (appearance (color): black; appearance (form): liquid; carbon: 40–50%; nitrogen: 3–6%; sulfur: ≤3%; residue on evaporation: 0.8–1.2 mg/mL).

Stock commercial GO dispersions, at concentrations indicated by the suppliers, were diluted in the ultrapure water (HPLC grade, Lichrosolv LC-MS water, Merck, Darmstadt, Germany). Four GO preparations were firstly shaken several times, and 1.4 mL (GO1), 1.75 mL (GO2), 7 mL (GO3) and 7 mL (GO4) were transferred to a 75 mL beaker and then dispersed up to 50 mL of the final volume with ultrapure water at the final GO concentration of 140 µg/mL. 

### 2.2. Graphene Oxide Sonication

The beakers were placed in water bath at 4 °C and sonicated directly by Ultrasonic Processor UP200St (Hielscher Ultrasonics GmbH, Teltow, Germany) using a sonotrode S26d14 immersed in the central point of dispersion (140 µg/mL of GO). The parameters of sonication were as follows: volume of 50 mL, power 50 W, time: 6 × 5 min with intermittent breaks (for cooling down to 15 °C) to reduce the loss of substance and a possible thermal reduction in GO. The sonication frequency was automatically modified according to power transmitted to dispersion (50 W).

### 2.3. Graphene Oxide Dilution with Bovine Serum Albumin (BSA)

After the last cooling of sonicated GO, the samples were combined with water dispersion of BSA (622.17 mg/L) to coat GO particles. Earlier, BSA (99.9% BSA, BLIRT, Poland https://blirt.pl (accessed on 12 October 2023), product not available anymore) as a dry substance was dissolved in ultrapure water (3.733 g/L, 55.995 mg of BSA in 15 mL of ultrapure water), stirred for 15 min at room temperature, filtered once with a filtering paper and then diluted 6× in 75 mL of ultrapure water. BSA dispersion (90 mL) was then combined with 50 mL of each GO dispersion, and mixed for 15 min with care taken to avoid foaming. 

### 2.4. Graphene Oxide–Bovine Serum Albumin Dilution with Krebs–Henseleit Solution

Graphene oxide–bovine serum albumin (GO-BSA) dispersions were combined with modified Krebs–Henseleit (KH) solution (1 volume unit of GO-BSA dispersion, i.e., 140 mL with 4 volume units of Krebs–Henseleit solution, i.e., 560 mL) and mixed for 15 min on a magnetic stirrer. Krebs–Henseleit solution reagents were dissolved in and reduced by 20% volume of ultrapure HPLC water (Lichrosolv LC-MS, Merck, Darmstadt, Germany) to obtain the final electrolyte concentrations with BSA dispersion. The final concentrations were as follows: all types of GO: 10 µg/mL, BSA 80 mg/mL; NaCl (Avantor Performance Materials Poland, Gliwice, Poland cat no 794121116 pure for analysis) 124 mM/L, KCl (Avantor Performance Materials Poland, Gliwice, Poland cat no. 739740114 pure for analysis) 4.2 mM/L, NaHCO_3_ 15 mM/L (Avantor Performance Materials Poland, Gliwice, Poland, cat no 810530115 pure for analysis), anhydrous glucose (Avantor Performance Materials Poland, Gliwice, Poland, cat no. 459560117 pure for analysis) 5.6 mM/L, MgSO_4_ anhydrous (Sigma-Aldrich, Poznań, Poland cat no. 1.06067 for analysis) 1.5 mM/L, KH_2_PO_4_ (Avantor Performance Materials Poland, Gliwice, Poland cat no. 742020112 pure for analysis) 1.2 mM/L, 99+% pure sodium pyruvate (Acros Organics, Belgium, now Thermo Scientific Chemicals, Hague, The Netherlands, cat no. A11148.18) 5.0 mM/L and CaCl_2_ anhydrous (Avantor Performance Materials Poland, Gliwice, Poland, cat no 874896118 pure for analysis) 1.5 mM/L. All Krebs–Henseleit buffer reagents were of analytic grade. The concentrations in the final Krebs–Henseleit solution were as follows: Na^+^ 144 mM/L, Ca^2+^ 1.5 mM/L, Mg^2+^ 1.5 mM/L, K^+^ 5.4 mM/L, Cl^−^ 131.2 mM/L, H_2_PO_4_^−^ 1.2 mM/L, SO_4_^2−^ 1.5 mM/L, glucose 5,6 mM/L, HCO3^−^ 15 mM/L.

### 2.5. Graphene Oxide Particle Size Analysis

The particle size, zeta potential (Z-potential) and conductivity measurements were performed using Zetasizer Nano ZS (Malvern Instruments, Malvern, UK) equipped with a standard He-Ne laser (633 nm). A non-invasive backscatter method (detection at 173° scattering angle) was used. Samples of the GO dispersions (1 mL) were placed in polystyrene cuvette (GO particle size measurements) or injected into the folded capillary zeta cell digit (zeta potential determination), and 3 (three) consecutive measurements were taken at 37 °C. An equilibration time of 120 s was set to ensure adequate warming before the measurement was started. An automatic measurement duration setting was used, with an automatic measurement positioning and attenuation.

The size distribution charts were plotted as an averaged value of the performed measurements. All data for each particle size distribution chart represent at least 30 measurements (3 measurements per sample) of the particle populations in the GO preparation stages. Two methods of analysis provided by instrument software were used: a cumulate method to determine an averaged particle size value (Z-average diameter) together with polydispersity index (PDI) showing how reliable the size estimation is and, furthermore, general-purpose algorithm based on a non-negative least squares (NNLS) to obtain particle size distributions in the peak means analysis (defined for three main fractions). Data processing for peak means analysis was set for the normal resolution and three main fractions were calculated by the ZetaSizer Nano ZS software (version 8.02). The numeric data were used both as averaged results of the measurements and as the mean values for statistical analyses of single results.

The electrophoretic mobility of GO particles in the dispersions was recorded based on a combination of electrophoretic and phase analysis light-scattering (PALS) techniques. The obtained data were automatically converted by the equipment software to the zeta potential (ζ) values using Henry’s equation. Default instrument settings and automatic analysis were used for all measurements.

### 2.6. pH Measurement

pH measurements were performed with the HI9829 Multiparameter Meter (Hanna Instruments Inc., Smithfield, RI, USA) device. Measurements of pH were performed in dispersions purchased from manufacturers, after dilution to the concentration of 140 µg/mL, after sonication and in the final dispersion with BSA in Krebs–Henseleit-modified solution. The results were stored after 30 s of stabilization.

### 2.7. Transmission Electron Microscopy

Transmission electron microscopy (TEM) images were obtained using a Tecnai F20 X TWIN microscope (FEI Company, Hilsboro, OR, USA) equipped with field emission gun, operating at an acceleration voltage of 200 kV and aperture of 40. The images were recorded on the Eagle 4k HS camera (FEI Company, USA) and processed with TIA software (FEI Company, USA). Samples (6 μL) were placed on a copper grid covered with holey carbon film and air dried at room temperature before measurements. 

### 2.8. Cryogenic Transmission Electron Microscopy

Cryogenic transmission electron microscopy (CRYO TEM) images were obtained using a Tecnai F20 X TWIN microscope (FEI Company, Hilsboro, OR, USA) equipped with field emission gun, operating at an acceleration voltage of 200 kV. Images were recorded on the Eagle 4k HS camera (FEI Company, Hilsboro, OR, USA) and processed with TIA software (FEI Company, USA). Specimen preparation was carried out by vitrification of the aqueous solutions on grids with holey carbon film (Quantifoil R 2/2; Quantifoil Micro Tools GmbH, Grosslobichau, Germany). Prior to use, the grids were activated for 15 s in oxygen plasma using a Femto plasma cleaner (Diener Electronic, Ebhausen, Germany). Cryo samples were prepared by applying a droplet (3 µL) of the solution to the grid, blotting with filter paper and rapid freezing in liquid ethane using a fully automated blotting device Vitrobot Mark IV (FEI Company, Hilsboro, Ore, USA). After preparation, the vitrified specimens were kept under liquid nitrogen until they were inserted into a cryo-TEM-holder Gatan 626 (Gatan Inc., Pleasanton, CA, USA) and analyzed in the TEM at −178 °C. 

### 2.9. Atomic Force Microscope Analysis

The morphology of the GO surfaces was characterized by an atomic force microscope (AFM). AFM images were obtained using a MultiMode with Nanoscope IIId controller, (Veeco, New York, NY, USA) AFM equipped with a piezoelectric scanner with a scan range of 10 × 10 µm^2^. The imaging of samples was conducted in the tapping mode in ambient air conditions at a scan rate of 1 Hz using etched silicon probes (TESP, BRUKER) of nominal spring constant 42 N/m and operating at a resonant frequency of 320 kHz. All samples were imaged at room temperature (RT). We used the Spin150 wafer spinner (Semiconductor Production Systems) under the following conditions: on the glass, at RT in air, 400 rpm, 60 min (AFM next day). Cover glasses were used (13 mm in diameter) and cleaned in acetone and in the plasma cleaner with oxygen (purity 6.0).

### 2.10. Functional Groups Determination

**Figure 1:**
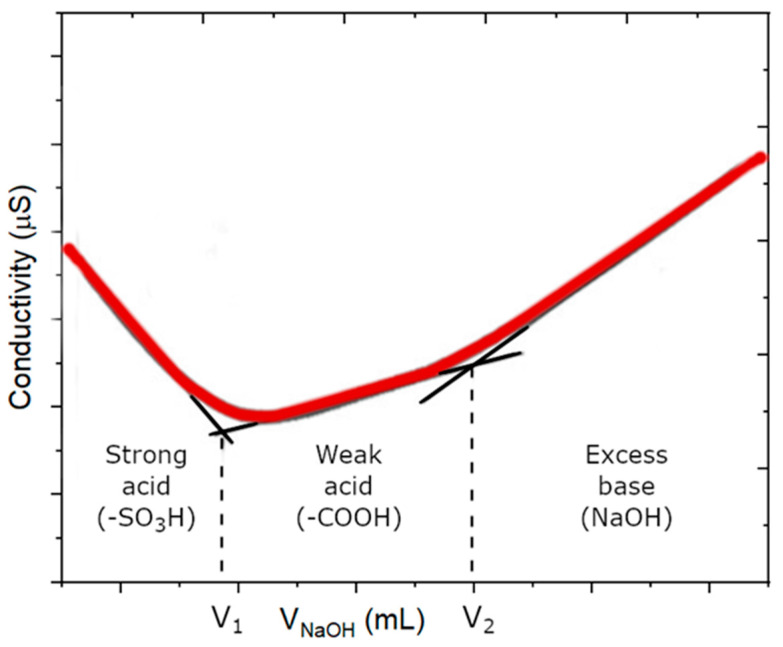
Typical course of the conductometric titration curve of GO dispersions with two distinct subcourses corresponding to the two relevant functional groups significantly differing in acidity, i.e., sulfonic (-SO3H) and carboxylic (-COOH) groups.

Sulfonic (-SO3H) and carboxylic acid (-COOH) content determination was performed by a conductometric titration (SevenCompact S230-Kit, Mettler-Toledo, Columbus, OH, USA) [23,24]. Briefly, dispersions containing 0.005 g of GO in 50 mL of 0.001 M NaClaq were titrated with 0.01 M NaOH at 20 °C using an automatic titrator (TitroLine 7000, SI Analytics, Mainz, Germany). Typical titration—presented as a function of conductivity (µS cm^−1^) versus VNaOH (mL)—covered two distinct subcourses, which were attributed to the presence of strongly acidic groups (-SO3H) and weaker acids (-COOH) (Figure 1).

Hence, the carboxyl content in the samples is given by Equation (1):

(1) $$\left[COOH]=\frac{(\left(V2-V1\right)\times CNaOH}{m\;nanoC}\;(mmol\;g^{-1}\right)$$

where V1 and V2 are the volumes of NaOH(aq) solutions corresponding to the two distinct inflection points on the titration curves (mL), C_NaOH_ is the exact NaOH(aq) concentration (mol L^−1^) and m_nanoC_ is the dry weight of the GO sample (g).

The sulfonic groups content in the samples is given by Equation (2):

(2) $$\left[-SO3]=\frac{\left(V1\times CNaOH\right)}{m\;nanoC}\;(mmol\;g^{-1}\right)$$

### 2.11. Statistics and Data Analysis

The results of single measurements were presented as mean and standard deviation (SD) and standard error on the plots. For statistical comparisons of GO samples, at the same stage of preparation, the Kruskal–Wallis ANOVA test was used to compare four groups representing four types of GOs. When the differences were significant, the Dunn’s test was used for post hoc analysis to compare the groups in pairs (Dunn’s test for independent samples). When measurements were compared in the consecutive stages, the Wilcoxon matched pairs test was used for dependent samples and mean values of the single measurements were used for statistic calculations. The differences were considered significant when *p* < 0.05. All the statistic calculations were performed using Statistica 12 (StatSoft, Kraków, Poland) and Origin Pro 2023 (Origin Lab, Northhampton, MA, USA) software. The averaged particle size distribution charts were analyzed separately.

## 3. Results

### 3.1. Graphene Oxide Water Dispersions Stock Characteristics

**Figure 2:**
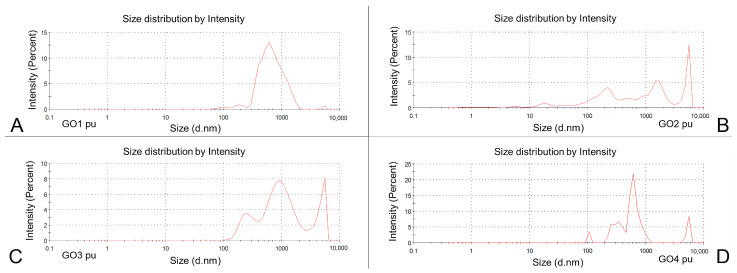
Size distribution by intensity, charts for purchased dispersions of GO samples: GO1pu (**A**), GO2pu (**B**), GO3pu (**C**), GO4pu (**D**). Data shown as averaged results of 30 measurements in 10 samples. Intensity expressed in percentages represents the percentage of given particle population in the entire particle population of the analyzed sample; size represents hydrodynamic particle diameter expressed in nm.

Upon visual inspection, GO dispersions were black in the original concentrations, rather thick and jelly-like in the case of GO1, more liquid-like in the case of GO2 and water-like in the case of both GO3 and GO4. It was impossible to see any precipitate in a bottle of GO1, a short time was required to notice granular sediments in bottles with GO3 and GO4 and the longest time was needed to notice sludge in bottles with GO2. When dispersed to the tested concentrations, GO3 and GO4 were black or grey-black, but GO1 and GO2 were yellowish-brown and transparent. These differences can confirm the higher oxygenation in GO1 and GO2 and lower oxygenation in GO3 and GO4. The particle size distribution charts for the averaged measurements of the original dispersions are presented in Figure 2. The particle sizes were assessed using two methods: with a Z-average analysis with PDI and with three peak means (the particle size distribution) to better observe the percentage distribution of the sizes in the particle fractions.

**Figure 3:**
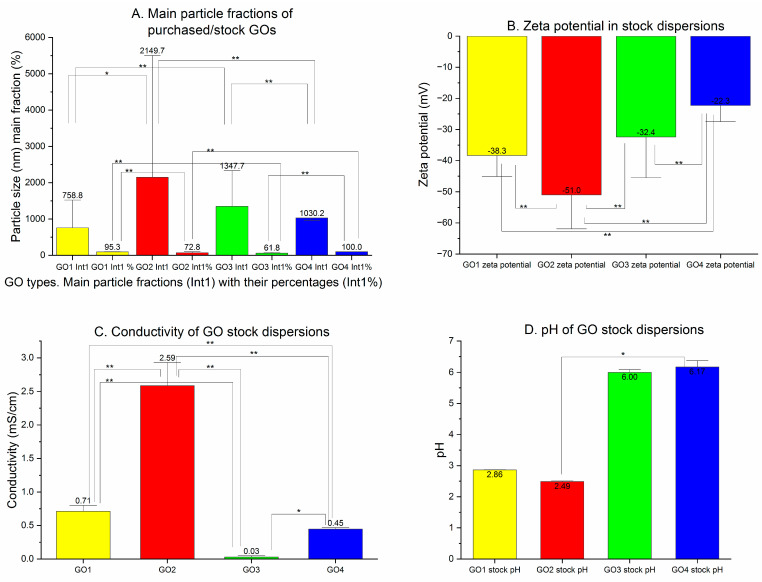
Stock dispersions of GO. (**A**) The mean particle size of the most frequent fraction (Int1) presented together with the percentage of the fraction in peak means analysis (Int1%) (results expressed as mean values of the single measurements). (**B**) Zeta potential of the original dispersions expressed in mV. (**C**) The conductivity of the original dispersions of GO in mS/cm. (**D**) pH measured in the original dispersions of GO (* *p* < 0.05; ** *p* < 0.01).

The GO1 particles were measured to have a Z-average of 3557 nm with a high PDI of 0.916. In the pooled particle population analysis (Figure 2A), the main peak was found at approximately 600 nm with the other particle populations at 100, 200 and 6000–7000 nm. Moreover, a trace of detected particles was noted below 100 nm and above 2000 nm. The peak means analysis revealed the domination of the 740 nm particles (Table 1). The GO2 particles had a Z-average equal to 4363 nm, with a lower PDI: 0.668. Their population analysis (Figure 2B) showed several peaks, marked at 20, 200, 500, 1000, 2000 and 6000 nm. In the distribution analysis, there were three almost equally frequent fractions observed between 188 and 5119 nm. The GO3 Z-average diameters were the lowest among all of the purchased GO dispersions: 1077 nm with PDI 0.717. The population analysis showed three clearly marked particle fractions at 200–300, 900 and 5000–6000 nm with a long trace of smaller particles below 100 nm (Figure 2C). In the peak means analysis, the main GO3 particle fraction was measured at 1049 nm (Table 1). The GO4 measurements were high in terms of the Z-average: 3386 nm with PDI 0.844. The main particle populations were around 100–200, 200–300, 300–400, 600 and 5500 nm when the pooled results were analyzed. The highest diameter found in the peak means analysis of the GO4 was the most frequent (657.3 nm—56.7%) (Figure 2D, Table 1). In the case of GO4, we found an important difference between the mean particle sizes calculated from single results and the averaged results (pooled particle population). All 10 samples presented a single particle fraction (1030.2 nm) when analyzed separately (as means of single measurements), but in the averaged results (Table 1), more particle fractions were seen. The difference is important in terms of the homogeneity of the nanoparticle population. Mean values for the dominant particle fractions found in single exported results from stock GO dispersions are presented in Figure 3A, together with the percentage of the dominant particle fraction in the whole particle population from the distribution analysis.

The stability of the dispersions was assessed with Z-potential measurements performed at every stage of the GO preparation. The zeta potential of the original dispersions is presented in Figure 3B. The Z-potentials of GO1, GO2 and GO3 were in the stability zone (−30.00 mV > Z-potential > +30.00 mV) with their absolute values > 30 mV, whereas the GO4 zeta potential was higher (−22.27 mV) and beyond the stability zone. All zeta potentials measured were negative, suggesting a negative charge of the particles.

In Figure 3C, we present the results of the conductivity measurements of the purchased dispersions of GO tested in the study. The highest conductivity was observed in GO2 dispersion (2.587 mS/cm) and the lowest in GO3 dispersion (0.030 mS/cm). Mean values of the conductivity measured in GO1 and GO4 presented the intermediate results. In Figure 3D, pH measurements are presented for the studied GO dispersions.

### 3.2. Graphene Oxide Dilution

The particle sizes of diluted dispersions are shown in Figure 3. Data for the whole particle populations expressed in numbers are shown in Table 1. 

**Figure 4:**
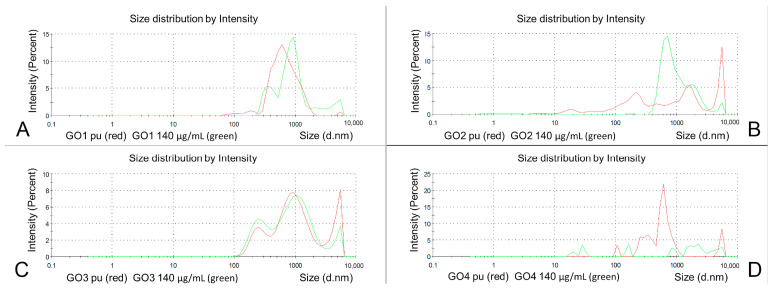
Size distribution by the intensity of dispersions of GO (red) and diluted dispersions in ultrapure water in a concentration of 140 µg/mL (green). GO1 (**A**), GO2 (**B**), GO3 (**C**), GO4 (**D**). Charts represent the averaged (pooled) results of 30 measurements in 10 samples.

The diluted dispersion of the GO1 presented a significant decrease in the particle size to 37% of the initial value of Z-ave (*p* < 0.0001), with a PDI reduction to 44% (*p* < 0.0001). In the averaged analysis of GO1 pooled samples, after dilution, three main peaks could be observed at 300–400, 900 and 6000 nm, with a long trace of small nanoparticles present below 100 nm (Figure 4A in green). The distribution analysis results are shown in Table 1. The diluted GO2 particles presented a size (Z-average) decrease down to 40% of the initial value (*p* < 0.0001), with a PDI decrease to 91% (*p* = ns). In the pooled GO2 population analysis (Figure 4B in green), the number of particle population peaks was reduced to three (at 700, 2000 and 5500 nm). The diluted GO3 also presented a decreased Z-average to 71% of the initial value (*p* < 0.0001) and a lowered PDI to 78% of the original value (*p* < 0.01). The averaged analysis of the whole GO3 particle population showed three main peaks with an increased number of particles between 200 and 300 nm and a decreased number of particles at 1000 and 5000–6000 nm (Table 1 and Figure 4C in green). After dilution, GO4 showed a significantly increased Z-average (776%, *p* < 0.001), while the decrease in PDI was not significant (from 0.844 to 0.758, *p* = ns), and much larger particles were found in all three fractions in the averaged peak means analysis (Table 1). These results conform to the change in the GO4 particles charge after dilution with significant aggregation. As seen in Figure 4D (in green), in the averaged particle population analysis, from five to seven fractions of the particles with similar and lower intensities could be observed within a range between 20 and 6000 nm. Having these effects of GO4 dilution, we increased the number of tested samples, but the results of all 42 measurements were similar, suggesting particle aggregation (Z-average measurement) and a lack of peak means analysis due to the large particle diameters. The pooled particle population analysis revealed three main particle populations representing only 62.4% of all particles. (Table 1, Figure 4D).

**Figure 5:**
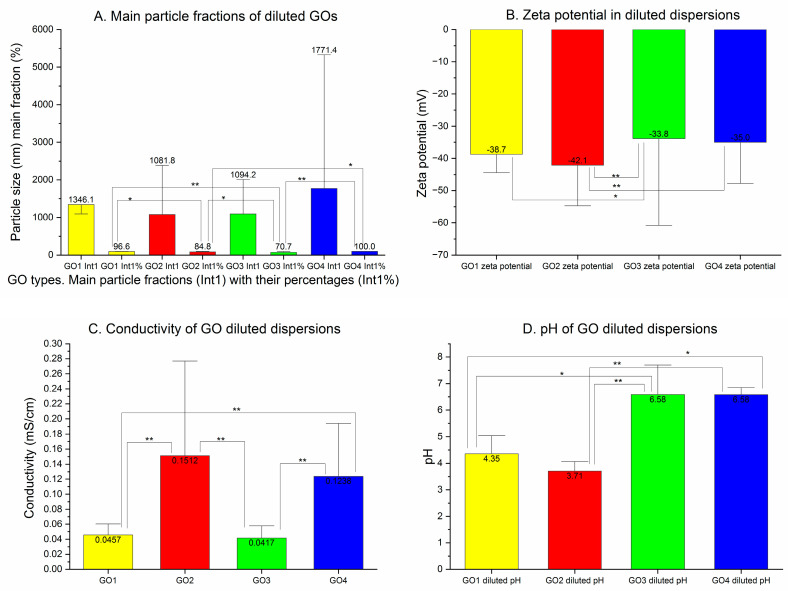
Diluted GO dispersions (140 µg/mL). (**A**) Mean particle sizes of the most frequent fractions are presented together with a percentage of the fraction in peak means analysis of single measurements. The GO4 bar represents only 15 measurements out of the 42 measurements performed. (**B**) Zeta potential of diluted GO dispersions (140 µg/mL) in mV. (**C**) Conductivity of the diluted GO dispersions (140 µg/mL) expressed in mS/cm. (**D**) pH measured in diluted dispersions of graphene oxides (140 µg/mL). (*p* < 0.05 *; *p* < 0.01 **).

Upon dilution, the mean dominant particle fraction size (Figure 5A) was not changed in GO1 (Int 1 *p* = ns, Int1% *p* = ns) and GO4 (Int1 *p* = ns, Int1% *p* = ns). The range of the Z-average in GO4 was between 82,470 and 2851 nm and, in Int1, between 19.8 and 5328 nm at this stage. In the diluted GO2 dispersion, the main particle fraction size was smaller (*p* < 0.02) and more frequent (the percentage increased significantly, *p* < 0.03). In the analysis of measurements of the diluted dispersion, only the percentage of the main particle fraction increased significantly (*p* < 0.01).

The zeta potential of the diluted dispersions was found to be negative and in the stability zone for all GOs (Figure 5B). With dilution, the zeta potential of GO1 and GO3 was not changed (*p* = ns), while, in GO2, the absolute value decreased (−51.01 mV before and −42.10 mV after dilution; *p* < 0.001). GO4 samples presented a substantial decrease in zeta potential from −22.27 mV to −35.01 mV (*p* < 0.0001). 

By dilution, an increase in pH was seen in all samples (for GO1: *p* < 0.03, GO2: *p* < 0.01, GO3: *p* = ns, GO4: *p* < 0.01), and conductivity decreased in GO1 (*p* < 0.0001), GO2 (*p* < 0.00001) and GO4 (*p* < 0.00001). In GO3, an increase in conductivity by 0.012 mS/cm was noted but was not significant (*p* = ns) (Figure 5C,D). 

### 3.3. Graphene Oxide Sonication

**Figure 6:**
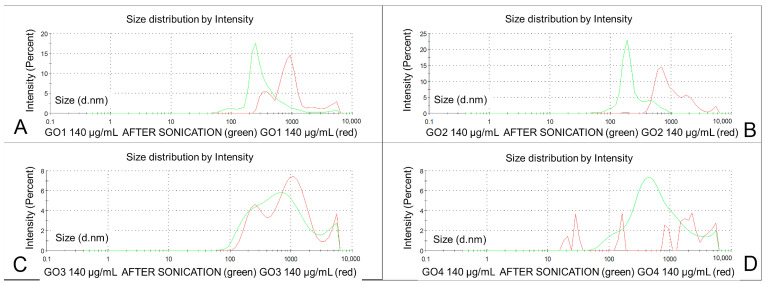
Size distribution by the intensity of four types of GO dispersions before (red) and after six sonication steps (50 W) (green). GO1 (**A**), GO2 (**B**), GO3 (**C**), GO4 (**D**). Charts represent the averaged results of 30 measurements in 10 samples.

The changes in the particle sizes after sonication are presented in Figure 6. The numerical representation of these charts is shown in Table 1. 

The sonicated GO1 measurements revealed a decrease in the Z-average to 46.7% of the initial value (from 1344 to 628 nm, *p* < 0.00001), but the PDI did not change significantly—from 0.412 to 0.518 (*p* = ns) (Table 1). In the distribution analysis of the averaged particle population, the dominant particle fraction was detected at 200–300 nm, but largest particle sizes were reduced in intensity at 5000–6000 nm, and the smallest detectable particle size was noted at 90–100 nm with a long trace of various particles between 0.4 and 90 nm (Figure 6A in green).

**Figure 7:**
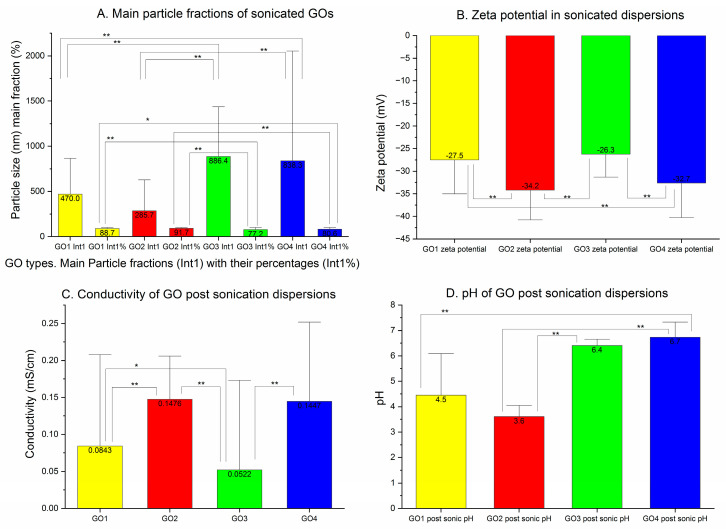
Sonicated dispersions of GO (140 µg/mL). (**A**) Mean particle sizes of the most frequent particle size fractions are presented together with a percentage of each fraction in peak means analysis. (**B**) Zeta potential of sonicated water GO dispersions (140 µg/mL) in mV. (**C**) The conductivity of the sonicated GO dispersions (140 µg/mL) expressed in mS/cm. (**D**) pH measured in sonicated GO dispersions (140 µg/mL (* *p* < 0.05; ** *p* < 0.01)).

The sonicated GO2 sample revealed a decrease in the particle size to 37.1% of the initial value (Z-average decreased from 1759 to 654.2 nm, *p* < 0.00001) while the PDI was lowered from 0.612 to 0.552 (*p* < 0.02) (Table 1). The main GO2 particle population in the averaged analysis was detected at 200 nm with a marked lower peak at 500 nm, with long tails of small amounts of particles below 100 nm and above 1000 nm (Figure 6B). The GO3 dispersions after sonication presented also decreased in particle size by Z-average from 768.8 to 532.1 nm (69.2% of initial value, *p* < 0.0001). The PDI for GO3 was lowered with sonication from 0.559 to 0.472 (*p* < 0.01) (Table 1). In the averaged population particle analysis, two main peaks were seen at 700 and 5000 nm with long tails of the particles below 70 and above 6000 nm (Figure 6C in green). For GO4, the decrease in the particle size was the most pronounced in the Z-average measurements (the decrease from 26,300 to 536.1 nm—2% of the initial size, *p* < 0.000001). The PDI was lowered significantly from 0.758 to 0.489 (*p* < 0.00001). In the averaged particle analysis, two particle fractions were seen at 400–500 nm and 6000 nm, instead of five to seven before the sonication of GO4 (Figure 6D in green). Post-sonication dominant particle size fractions (Int 1) of the sonicated GOs are presented in Figure 7A. 

The GO1 main particle fraction size in the peak means analysis decreased to 34.9% of the presonication value, (*p* < 0.0001) with a reduced percentage (88.73%, *p* < 0.05) (Figure 7A). In GO2, the main fraction was more frequent (91.7% of particle population, *p* = ns) and also smaller: 26% of the presonication value (*p* < 0.00001). GO3 presented a smaller reduction in the main fraction size to 81% of the presonication value, (*p* = ns) with a tendency toward a higher percentage of the main fraction in the entire particle population (77.2%, *p* = ns). In GO4, all the samples could be measured with both algorithms used (due to lower particle diameters of sonicated GO4). Post-sonication results presented a mean dominant Int1 fraction diameter of 838.4 nm (47% of the presonication result was restricted to only 15 available measurements, *p* = ns; other measurements were beyond the range of the device), with the percentage being 80.57% (Int1% significantly lower, *p* < 0.001). 

The absolute Z-potential values of all GO dispersions were reduced after sonication. The GO1 mean Z-potential increased from −38.72 mV before sonication to −27.51 mV after sonication (*p* < 0.0001). For GO2, the zeta potential was increased from −42.10 mV to −34.20 mV (*p* < 0.00001). The absolute value of the Z-potential of the sonicated GO3 was reduced from 33.85 mV to 26.26 mV (*p* < 0.01) and, in GO4, it was reduced from 35.01 mV to 32.65 mV (*p* < 0.05). The Z-potential for sonicated GO1 and GO3 was beyond the stability zone and negative. The Z-potential of sonicated water GO dispersions is shown in Figure 7B. All the dispersions were without any signs of aggregation.

After sonication, the conductivity of the GO water dispersions was increased in GO1, GO3 and GO4 and decreased in GO2 (Figure 7C). In GO1, it increased from 0.046 mS/cm to 0.084 mS/cm (182% of the initial value, *p* < 0.01); in GO2, the observed change was between 0.151 mS/cm and 0.148 mS/cm (decrease to 98% of the presonication value, *p* = ns). For GO3, the increase in conductivity from 0.042 mS/cm to 0.052 mS/cm (to 123.8% of presonication value) was not significant (*p* = ns). The increase in the conductivity of the sonicated GO4 dispersion was lower than in GO3—to 116.9% of the presonication value, from 0.124 mS/cm to 0.145 mS/cm (*p* = ns). 

The mean pH value measured after sonication decreased in GO1, GO2 and GO3 and increased in the sonicated GO4 dispersion (Figure 7D). For GO1 and GO2, pH reduction was observed from 3.71 to 3.61, and GO3 dispersion also presented a reduction in pH from 6.58 to 6.4. In the GO4 dispersions, on the contrary, an increase in pH was seen from 6.58 to 6.73. Furthermore, GO samples were studied in terms of a qualitative and quantitative assessment of the functional groups and so, after the sonication, proportions and concentrations of COOH and SO_3_H were changed and differences between the sonicated GO types were less pronounced. In all the studied GO dilutions after sonication, the content of the carboxylic group increased from twice to thrice as compared with the non-sonicated GO dilutions (Table 2). 

### 3.4. Graphene Oxide Dilution with Bovine Serum Albumin

**Figure 8:**
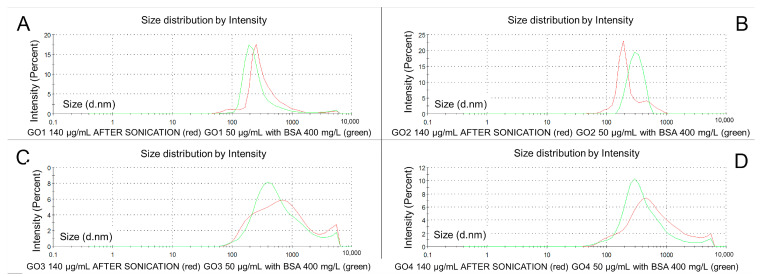
Size distribution in four types of GO dispersions after six sonication steps (red) and after adding BSA (green). GO1 (**A**), GO2 (**B**), GO3 (**C**), GO4 (**D**). Charts represent mean values for 10 samples measured 3 times each.

Figure 8 represents changes in the particle size distribution for GO dispersions before (red) and after combining with BSA (green). In this procedure, we in fact applied two changes: dilution (from 140 to 50 µg/mL) and a new GO-BSA interaction. All data are shown in Table 1.

In the particle population of the GO1 dispersions with BSA, the particle size (Z-average) decreased from 628 to 357.9 nm (to 57% of initial value *p* < 0.00001), and a lower PDI was seen in the BSA-GO dispersion (0.518 before and 0.38 after *p* < 0.0001) (Table 1). In the averaged population, the analysis of GO1 BSA dispersion presented one population of particles at 200 nm that was almost symmetrical (Figure 8A in green). The mean Z-average of GO2 decreased from 654.2 to 408.2 nm (62.4% of initial value, *p* < 0.0001) and PDI from 0.552 to 0.418 (*p* < 0.001). The graphic representation of the GO2 and GO2-BSA averaged particle population showed the rightward movement of the GO2-BSA curve, which was bell-shaped (peak at 300 nm) but single-peaked, with two trace tails on both sides (Figure 8B). GO3 changed particle sizes with the same pattern. The Z-average decreased in the GO3-BSA dispersion to 509.7 nm (95.7% of the initial diameter, *p* = ns) with the PDI changing from 0.472 to 0.391 (*p* < 0.001) (Table 1). In the averaged graphic analysis (Figure 8C in green), the GO3-BSA particle populations presented two main fractions: at 400 nm and 5000–6000 nm. The GO4-BSA mean Z-average was lowered from 536.1 to 441.8 nm (82% of the initial value, *p* = ns), with a PDI decrease from 0.489 to 0.448 (*p* = ns). In the graphic representation of the GO4-BSA particle size distributions, the dominant particle fraction was detected at 200 nm, with a smaller peak in the range of 5000–6000 nm. In all dispersions at this stage, only two particle fractions were detected in the particle size distribution analysis.

**Figure 9:**
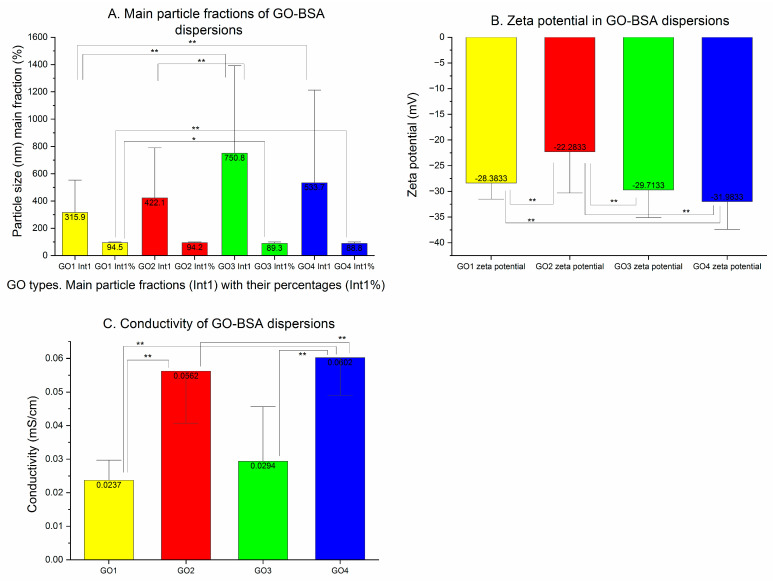
Sonicated GO dispersions with BSA. (**A**) Mean particle sizes of the most frequent fractions are presented together with a percentage of the fraction in peak means analysis and statistical analysis performed on single measurements. GO in concentration of 50 µg/mL and BSA in concentration of 400 mg/L. (**B**) Zeta potential of sonicated water GO dispersions with BSA in mV. (**C**) The conductivity of the sonicated GO dispersions with BSA expressed in mS/cm. (* *p* < 0.05; ** *p* < 0.01).

The dominant particle fractions at the BSA-GO stage revealed smaller particle sizes with higher percentages of the particle populations. In GO1 with the BSA sample, the dominant particle fraction size by intensity had 315.88 nm (67% of post-sonication size, *p* = ns) and 94.47% of all detected particles (Figure 9A).

The GO2-BSA dominant particle fraction size by intensity was increased from 285.7 nm to 422.1 nm (147% of initial size, *p* < 0.01) with an increased percentage in the general population (94.2%, *p* = ns for the change). GO3 with BSA had a smaller particle size of the dominant particle fraction by intensity: Int 1 (750.83 nm after combining with BSA, *p* = ns for the change), with its increased percentage 89.34% (*p* < 0.001 for the change). In GO4 measurements, the dominant particle fraction was seen in 88.85% of the particle population (earlier 80.57%, *p* < 0.01), and the particle size by intensity was also smaller (533.7 nm after combining with BSA—63.6% of initial size, *p* = ns). At this stage, Z-potential and conductivity measurements were made and the results are shown in Figure 9B,C.

The Z-potential for GO1-BSA was higher in absolute value than sonicated GO1 (before 27.51, after 28.38—change to 103% of the initial value, *p* = ns). The GO2-BSA absolute value of the zeta potential decreased from 34.20 to 22.28 (65% of the initial value, *p* < 0.00001). The GO3-BSA dispersion changed the zeta potential absolute value from 26.26 to 29.71 (113% of the initial value, *p* < 0.001). The GO4-BSA zeta potential mean value changed from −32.65 to −31.98 mV (the absolute value changed to 98% of the initial value, *p* = ns) (Figure 9B). In turn, electrical conductivity decreased in all of the GO-BSA dispersions due to the dilution and amalgamation of GO nanoparticles with the insulating BSA macromolecules. In the GO1-BSA dispersion, the conductivity was changed from 0.084 to 0.024 mS/cm (28% of initial value, *p* < 0.00001), and in GO2-BSA, it was lowered from 0.148 to 0.056 mS/cm (38% of the initial value, *p* < 0.001). For GO3-BSA, the observed conductivity change was between 0.052 and 0.029 mS/cm (55% of the initial value, *p* = ns) and for GO4-BSA, from 0.145 to 0.60 mS/cm (41% of the initial value, *p* < 0.001) (Figure 9C).

### 3.5. Graphene Oxide Dispersions with Bovine Serum Albumin and Krebs–Henseleit Solution

**Figure 10:**
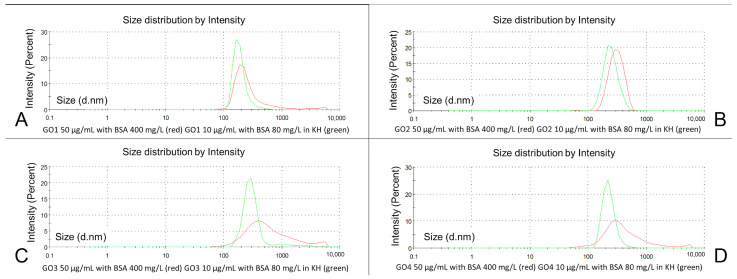
Size distribution by the intensity of sonicated dispersions with BSA (red) and dispersion of GO with BSA and Krebs–Henseleit (KH) buffer (green) for each analyzed GO. GO1 (**A**), GO2 (**B**), GO3 (**C**), GO4 (**D**). Charts represent mean values for 10 samples measured 3 times each.

Already having ultrapure water in the GO-BSA dispersion, the volume of water dedicated to the preparation of the crystalloid solution for the KH buffer was reduced by 20% at the stage of dissolving the KH reagents. Importantly, adding the crystalloid solution with a higher-than-isotonic reagents concentration (20% volume water reduction) to the BSA-GO water dispersions did not cause GO particle aggregation. The particle populations in the final GO-BSA-KH dispersions became monodisperse (GO1, GO4), and almost monodisperse (Figure 10A) with a low number of particles smaller than the mean particle diameter observed (GO2), or bigger than the main particle fraction as in GO3 in averaged particle size analysis (Table 1: peak 2 average int: 1839 nm). The single results analysis revealed significant increases in the main particle populations percentages and significant decreases in the main fraction particle sizes in all GOs. Unexpectedly, in the Z-ave results, not all particles were smaller in final dispersions. Moreover, we found the differences in particle population sizes assessed with the cumulate method (Z-Ave and PDI) and non-negative least squares algorithm (NNLS) in monodisperse particle populations (GO1 and GO4), where one could expect similar results (Table 1). In detail: GO1 diluted in BSA and isotonic high-ionic-strength crystalloid solution increased the particle size by Z-average from 357.9 to 421.1 nm (*p* < 0.04), with the final PDI equal to 0.385 (*p* = ns) (Figure 10, Table 1). 

The peak means analysis showed a decrease in the single-particle fraction size to 193.6 nm (67.7% of the initial value *p* < 0.05) (Table 1). The same pattern of changes was observed in GO4, where a single population of particles was also observed and a significantly lowered particle size was noted in the peak means analysis (234 nm, *p* < 0.00001) on the contrary to the Z-average, where an increase in the particle size was observed from 441.8 nm to 490.1 nm (*p*-ns). GO2 diluted with BSA in the KH solution decreased the particle size in the Z-average from 408.2 nm to 382.6 nm (*p* = ns) and PDI to 0.363 (*p* < 0.05). GO3 presented a decrease in particle size in Z-average measurement (to 440.2 nm—86.3% of initial size; *p* < 0.04) but the PDI was not changed (0.406; *p* = ns). 

**Figure 11:**
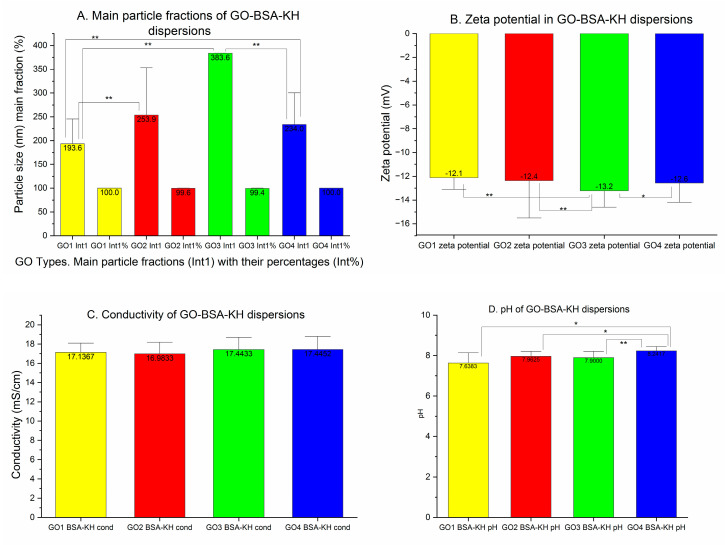
Sonicated GO dispersions with BSA in KH buffer. (**A**) Mean particle sizes of the most frequent fractions are presented together with the percentage of the fraction in peak means analysis. (**B**) Zeta potential of sonicated water GO dispersions with BSA in KH buffer in mV. (**C**) Electrical conductivity of the sonicated GO dispersions with BSA in KH buffer expressed in mS/cm. (**D**) pH values of the sonicated water GO dispersions with BSA in KH buffer (* *p* < 0.05; ** *p* < 0.01).

In GO2, the dominant particle fraction Int1 was reduced in size from 422.1 nm to 253.9 nm (to 60.1% of the initial size, *p* < 0.001), while its percentage was increased to 99.61% (*p* < 0.01). The size of the dominant particle fraction in GO3 was reduced in the KH solution to 383.59 nm (*p* < 0.01), also with an increase in the percentage (99.44%, *p* < 0.0001). GO1 and GO4 particles were homogenous in all the measured samples (Table 1). Under the high ionic strength of the KH buffer, an increased number of particles in single, dominant fractions with bell-shaped peaks for all GO dispersions was observed in the averaged particle population analysis. This finding was supported by low PDIs in all analyzed dispersions. The Z-potentials of the GO-BSA dispersions in the KH buffer were lower than in GO with BSA without the KH buffer. The absolute value of the zeta potential for GO1 was lowered to 42.6% of the initial mean (*p* < 0.00001). In GO2, GO3 and GO4, the reductions in mean absolute values were, respectively, 55.4% (*p* < 0.00001), 44.4% (*p* < 0.00001) and 39.3% (*p* < 0.00001). None of them were in the stability zone as in the simple ionic solution. (Figure 11B). The observed result of the decrease in absolute value of the zeta potential was caused by the presence of monovalent and divalent cations together with an increased ionic strength [25]. At a higher ionic strength, the electric double layer of the particle in the solution is compressed; therefore, we observe lower absolute values of the zeta potential. This effect is intensified at a high pH (above the isoelectric point of the BSA) by bivalent cations and, on the contrary, it is reduced in the presence of monovalent cations. In turn, electrical conductivity, as expected for the KH buffer, was increased significantly due to the presence of electrolytes. Mean values are shown in Figure 11C. No significant differences were found between the forms of GO concerning the conductivity. The pH values were close to 8.00 and were lower in GO1, GO2 and GO3 than in GO4 (Figure 11D). No signs of aggregation were seen in any dispersion.

### 3.6. Graphene Oxide as Analyzed by Transmission Electron Microscopy and Cryo-Transmission Electron Microscopy

**Figure 12:**
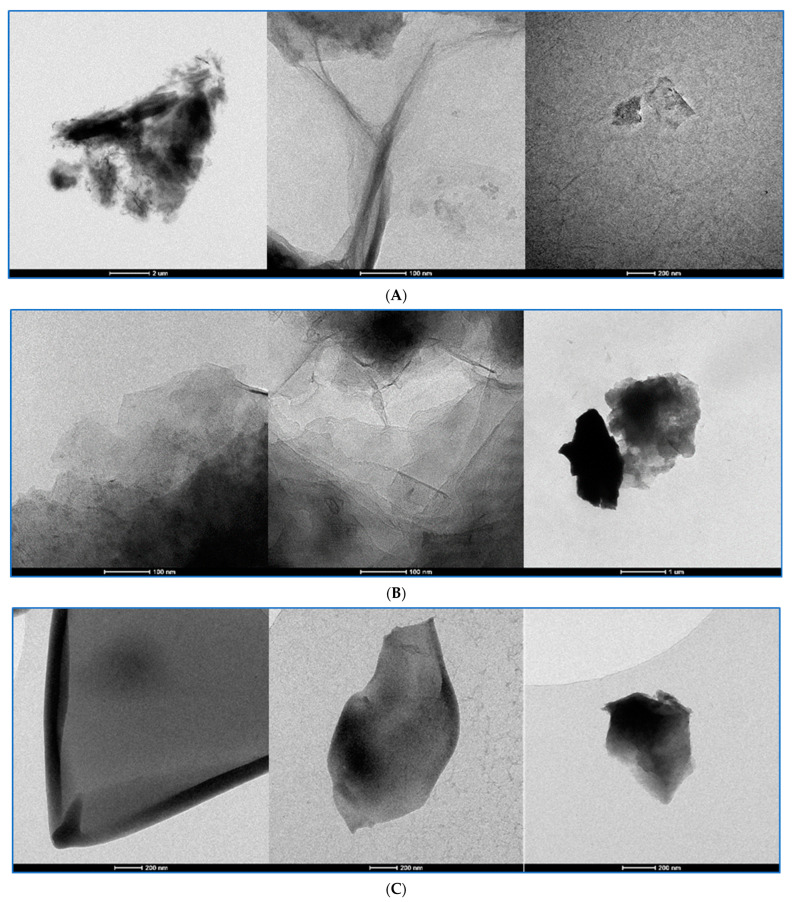
The GO1 at three chosen stages of preparation visualized with TEM and cryo-TEM. Stage and calibration description under the pictures. (**A**): GO1 stock dispersion in TEM. The calibration bars in the bottom of the pictures represent, from left to right, 2 µm; 100 nm; 200 nm. (**B**): GO1 dispersion with BSA in TEM. The calibration bars in the bottom of the pictures represent, from left to right, 100 nm; 100 nm; 1 µm. (**C**): The GO1-KH-BSA dispersion visualized with CRYO-TEM. The calibration bars in the bottom of the pictures represent, from left to right, 200 nm; 200 nm; 200 nm.

**Figure 13:**
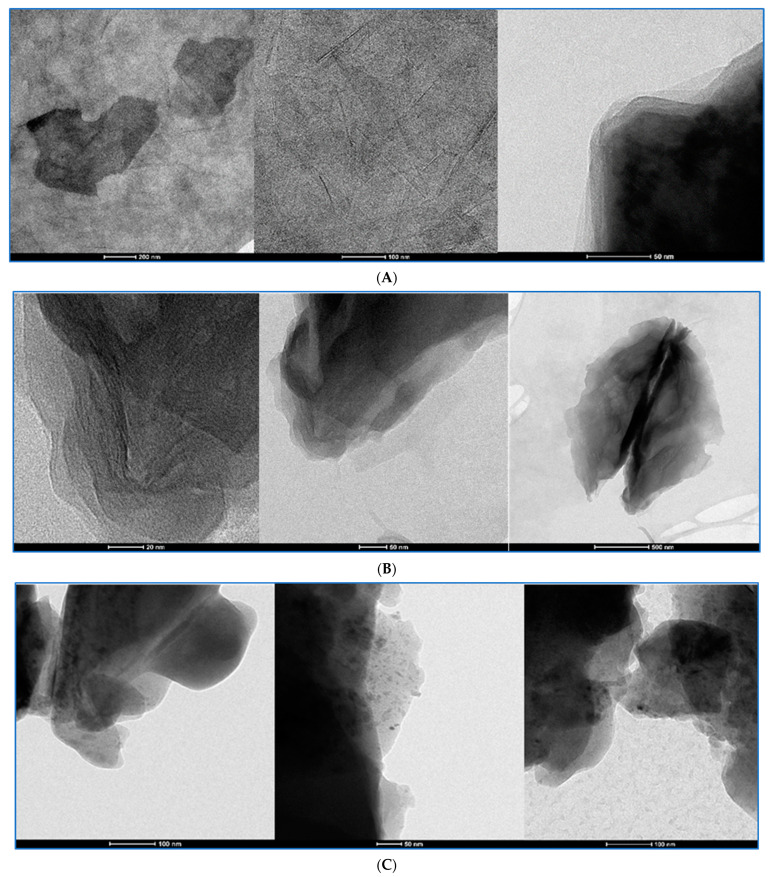
The GO2 at three chosen stages of preparation seen in TEM and with cryo-TEM. Stage description under the pictures. (**A**): GO2 stock dispersion The calibration bars in the bottom of the pictures represent, from left to right, 200 nm; 100 nm; 50 nm. (**B**): The GO2 dispersion with BSA. The calibration bars in the bottom of the pictures represent, from left to right, 20 nm; 50 nm; 500 nm. (**C**): The GO2-KH-BSA dispersion visualized with CRYO-TEM. The calibration bars in the bottom of the pictures represent, from left to right, 100 nm; 50 nm; 100 nm.

**Figure 14:**
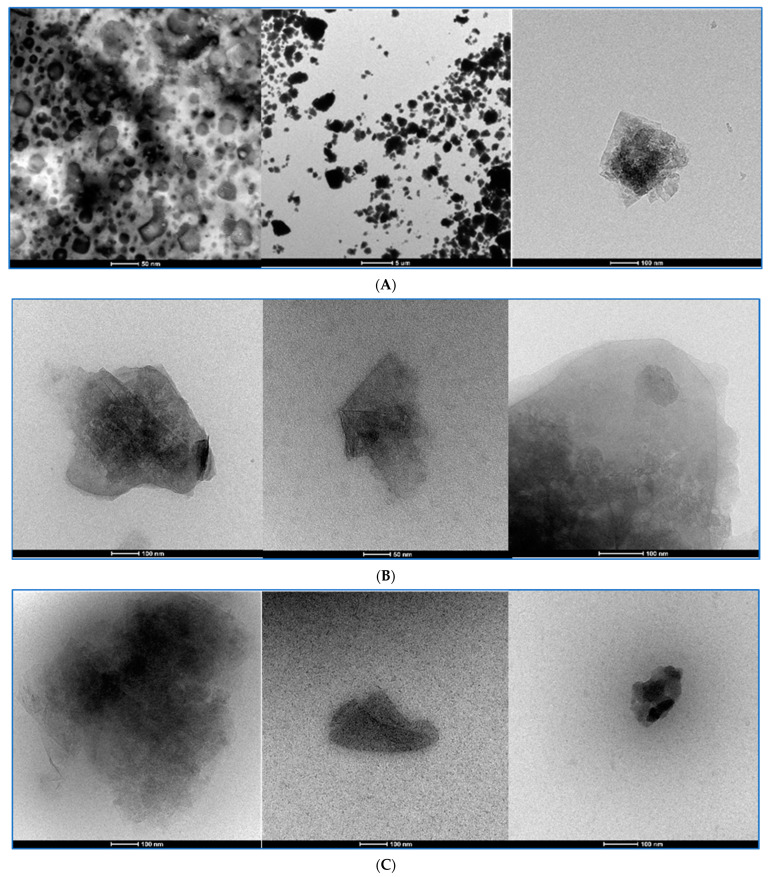
The GO3 at three chosen stages of preparation seen in TEM and with CRYO-TEM. Stage description under the pictures. (**A**): The GO3 stock dispersion visualized with TEM. The calibration bars in the bottom of the pictures represent, from left to right, 50 nm; 5 µm; 100 nm. (**B**): The GO3 dispersion with BSA visualized with TEM. The calibration bars in the bottom of the pictures represent, from left to right, 100 nm; 50 nm; 100 nm. (**C**): The GO3-KH-BSA dispersion visualized with CRYO-TEM. The calibration bars in the bottom of the pictures represent, from left to right, 100 nm; 100 nm; 100 nm.

**Figure 15:**
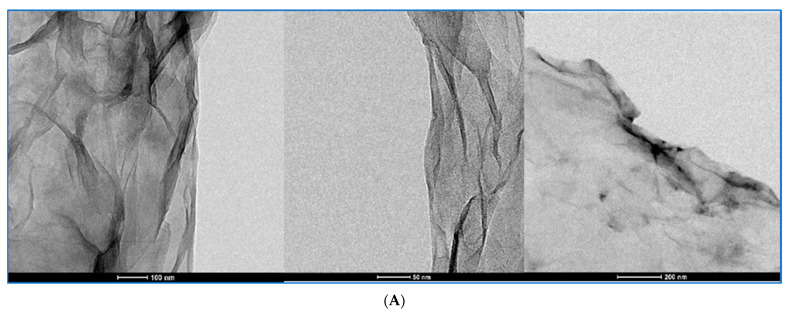

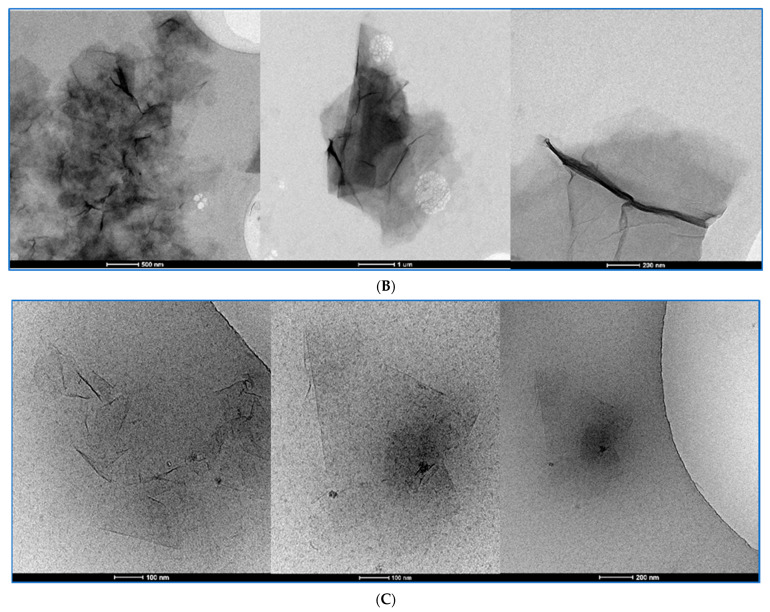
GO4 at three chosen stages of preparation visualized with TEM and cryo-TEM. Stage description under the pictures. (**A**): The GO4 stock dispersion visualized with TEM. The calibration bars in the bottom of the pictures represent, from left to right, 100 nm; 50 nm; 200 nm. (**B**): The GO4-BSA dispersion visualized with TEM. The calibration bars in the bottom of the pictures represent, from left to right, 500 nm; 1 µm; 200 nm. (**C**): The GO4-KH-BSA dispersion visualized with CRYO-TEM. The calibration bars in the bottom of the pictures represent, from left to right, 100 nm; 100 nm; 200 nm.

Graphene oxide dispersions were analyzed in TEM and cryo-TEM at three chosen preparation stages as the original dispersions, sonicated dispersions with BSA and the final Krebs–Henseleit dispersions with BSA. cryo-TEM was applied to avoid the crystallization of salts present in the KH solution in the last preparation step. Representative images are shown in Figure 12, Figure 13, Figure 14 and Figure 15. To compare changes in the particles during the preparation stages, each figure presents a GO in three dispersions. GO1 and GO4 were observed as single-layered preparations whereas GO2 presented as few-layer particles and GO3 as a densely packed granular form declared as 15–20-layer particles. 

For all GO samples, TEM imaging (Figure 12, Figure 13, Figure 14 and Figure 15) revealed a progressive individualization of GO flakes from ultrasonication-assisted as-made stock (sets A) via BSA-stabilized (sets B) to BSA-stabilized with counteracting in terms of stability and the presence of divalent inorganic cations (sets C). The individualization corresponded to the decreasing size of GO agglomerates dependent on the initial size and morphology of GO samples. After the addition of BSA and Krebs–Henseleit solution, uniformly co-dispersed BSA macromolecular amorphous nanoaggregates and inorganic nanocrystallites could be detected in all samples. It is important to note that sharp edges or facets were found for GO1, GO3 and GO4 while, after BSA-driven stabilization, mostly rounded particles could be indicated among the GO3 flakes. At the same time, the GO4 samples had the highest tendency to form wrinkles. This morphological parameter cannot be neglected from a biological point of view as defect- [26] and functionalization-rich [27] wrinkles might represent the hot spots for further enzyme degradation [28]. Having in mind hope for creating new anticancer drugs with GO as the drug platform, we have to admit that well-established models for predicting toxicity of the xenobiotics [29] (like QSTR) do not propose practical tools or pathways for the degradation of structures like GO. Improving the stability of the particle dispersions and its structure and preventing forming large agglomerates can surely help in activating well-known ways [30] of performing GO bioremoval. GO deagglomeration was also found to extensively occur upon the adsorption of low-molecular compounds from aqueous solutes [31]. The tendency toward wrinkling was also found for graphene sheets at the polar and non-polar interfaces [32]. At the same time, GO rich in sharp edges and facets showed an increased cytotoxicity in vivo [33]. In summary, to realistically portray GO flakes, one must consider statistical morphological features from the micro- to nanoscale with an accompanying quantitative and qualitative analysis of physicochemical functionalization, including the complex nature of the GO/nanoparticle/liquid interface. 

The cryo-TEM method helped us to visualize the BSA-GO interactions that were different among the types of GOs. In the case of GO2 and GO4, both cryo-TEM and TEM at the stage of BSA-GO complexes revealed the granular additional structures on the particle surfaces. The pH increase and introduction of bivalent cations have not modified this picture in the case of GO2 but they were less expressed in GO4. 

## 4. Discussion

**Figure 16:**
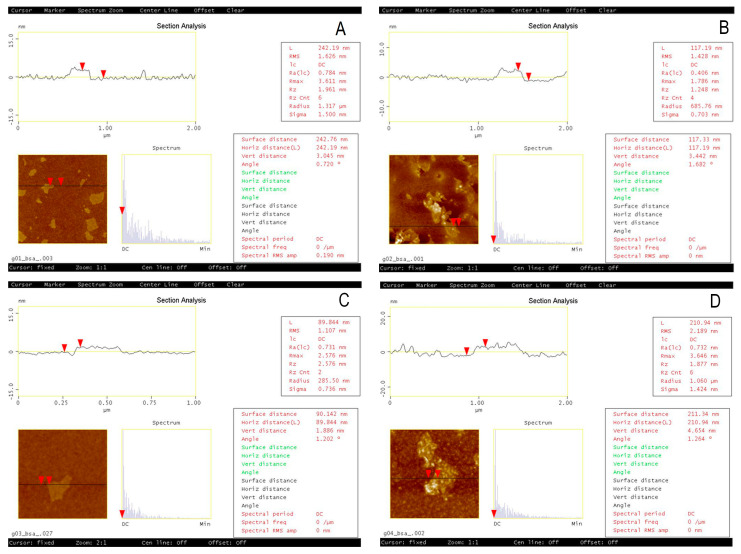
The atomic force microscope (AFM) of four types of GO with BSA: (**A**): GO1, (**B**): GO2, (**C**): GO3, (**D**): GO4. The granules of the BSA are easily visible in GO2 and GO4, but not in the case of GO1 and GO3. The red triangles depict the points of the measurements both for vertical and horizontal directions. The vertical distance depicted in the measurement result area refers to the thickness of the GO particle.

The main objective of this work was to prepare and characterize isotonic GO dispersions stabilized with BSA to be used in biomedical organ perfusion studies. The analysis of GO-BSA interaction can be crucial for the creation of GO-based drug transport platforms, biosensors and contrast components that were already proposed for in-blood use (Figure 16). Graphene oxide (GO) forms more stable dispersions in water than pristine graphene or reduced GO [34,35]. The observed increased dispersion stability is dependent on the GO oxygen structural content.

Four types of different, commercially available GO were selected for this study to represent a low (GO1, GO2) and high (GO3) number of GO layers, low (GO3) and higher (GO1, GO2) oxygen content and modification of the sp^2^-carbon lattice (N-doped GO4 of low oxygen content). To investigate the particle population changes, we used three methods of particle size measurement and analysis (cumulate analysis; NNLS algorithm with comparison of single and averaged measurement results). Such an approach was aimed at a detailed analysis of particle populations sizes, as the particle diameter is important for the biological effects of nanoparticles.

The particle populations of the stock samples were measured and proved to be polydisperse with variable percentages of smaller and larger particles. Polydispersity indexes for all four initial GO samples were higher than 0.6. The particle size range was observed between 97.6 nm and 5119 nm. Only GO1 presented one dominant particle population (740.1 nm—95%) in the peak means analysis, with much lower percentages of two other particle fractions in both analyses (see Table 1). All GO4 samples were monodisperse in single triplicated measurements but this was not confirmed in the averaged measurement of the pooled particle population. Particle size is an important determinant of the biological effects of GO. Lin and colleagues [36] found that all GO particles increased cytotoxicity in murine neural stem cells culture at a concentration > 20 μg/mL. Larger GO particles (1 μm and 5 μm) were more toxic than smaller ones (400 nm and 700 nm). Both 417 nm- and 663 nm-GO intensified the self-renewal of the studied cells in the absence of growth factors, while GO of a flake lateral size of 4651 nm was more efficient in promoting cellular differentiation. Moreover, GO 417 nm increased the migration of neuronal stem cells by upregulating the expression of vinculin. In another study on mitochondrial activity [37], the particle size of GO as well as the time of exposure were crucial for the survival of two cell lines. The proposed mechanism of the observed cellular toxicity of GO was based on the intracellular reactive oxygen species (ROS) generation causing cellular membrane and mitochondrial damage, finally resulting in apoptosis via the transforming growth factor beta pathway [38]. 

In general, the GO toxicity depends on individual particle features like size [39], surface charge [40], particle shape [41], the presence of functional groups [42] and the number of layers, as well as the aggregation state [30]. Therefore, the preparation of stable dispersions [43] under an isotonic ion concentration with a single-size particle population is critical for the investigation of nanoparticle effects on cells and tissues. 

All GO dispersions showed acidic pH, which was significantly lower in GO1 and GO2 when compared to GO3 and GO4. Electrical conductivity varied significantly among the types of GO, ranging from 0.03 up to 2.58 mS/cm. Zeta potential was found in the stability zone for GO1, GO2 and GO3 and beyond the stability zone for GO4. The stability zone in the zeta potential scale was above the absolute value of 30 mV. The higher the electrical charge, the stronger the particle repulsion and therefore the increased stability of the dispersion. In all samples in this study, the particle charge and zeta potential were negative.

Dilution was used to unify the aqueous dispersions before sonication to ensure similar energy transfer in comparable particle concentrations. The dilution degree was higher in GO1 and GO2 than in GO3 and GO4. As expected, it resulted in an increased pH due to the weakening of the face-to-face aggregation [44] in all types of dispersions. This pH increase was significant for GO1, GO2 and GO4, but not for GO3. A significant decrease in particle size, as measured by Z-average, was observed in GO1, GO2 and GO3 since dilution diminished particle interaction and lowered the interparticle contact and aggregation. Furthermore, GO as an anisotropic macromolecule is known to form ordered structures known as crystalline phases in dispersions with increasing concentrations of particles, where intense interaction creates organized structures that are so-called liquid crystals. According to Onsager’s theory, both dilution and an increased particle concentration together with a possible decreased diameter/thickness ratio could be responsible for a dispersion change from the nematic to isotropic phase and, therefore, changes in physical properties of the dispersion [45]. In our experiments, GO1 and GO2 were the types of particles that were most prone to such changes, having the highest percentage of single-layer or few-layer forms. 

In GO3 and GO4, the dilution degree was lower, with a lower absolute change in pH and two patterns of response to the dilution. There was a nonsignificant increase in pH and only a tendency toward a higher conductivity in the GO3 diluted dispersion, whereas a significant pH increase associated with massive particle aggregation was observed in the GO4 diluted dispersion. The zeta potential in the GO4 dispersion was within the stability zone only after the dilution, whereas in GO1 GO2 and GO3, it was within the stability zone before and after dilution, with an absolute value > 30 mV.

GO flakes are usually extracted from oxidized graphite blocks. Under well-defined and controlled conditions, sonication can give stable dispersion controlled only by electrostatic stabilization. Such a dispersion represents typical features of colloids [46]. Electrostatic repulsion of the separated GO particles is modified by sodium chloride, which can lead to aggregation and sedimentation. Bivalent cations such as calcium can further intensify the GO aggregation through creating bridges between the GO functional groups in separate flakes [47,48]. Sonication was also reported to enable layer control of the GOs during the processing of the graphite with varying energies. In Wang et al.’s study, changing the sonication power from 60 W to 300 W resulted in a reduction in the final graphene thickness from 4–10 layers down to a single-layer sample [49]. In our study, a significant decrease in particle size was detected after sonication in all GOs. The highest impact of sonication was observed in GO4, where the Z-ave particle size decreased to 2% of the pre-sonication particle diameter. In this case, a sediment observed after dilution was reintroduced to the dispersion, and the larger particles deagglomerated. All particle populations presented a significantly lower PDI together with a smaller size of the dominant particle populations, and these changes were significant in GO1 and GO2. At this preparation stage, the number of particle fractions decreased for the first time in GO3 and GO4, but this change was detected only in averaged particle size analysis.

**Figure 17:**
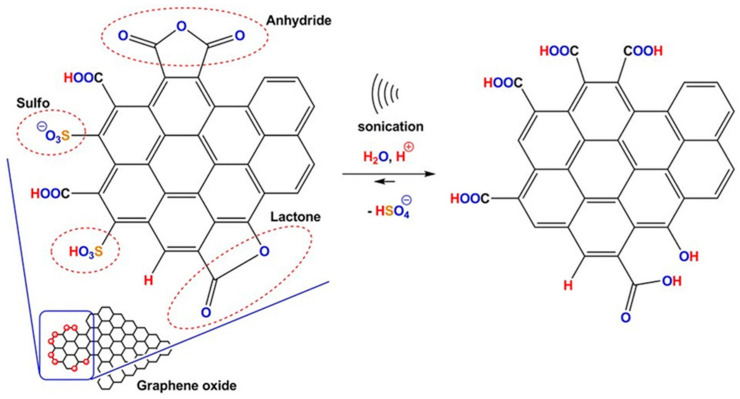
Illustration of hydrolysis of anhydride (and lactone) groups accompanied by desulfonation of graphene-oxide-based materials obtained by wet chemistry exfoliation. Dashed ovals surround the areas of the particle modifications.

Sonication also modified the GO particle chemical structure. The results of titration analysis confirmed changes in concentrations of COOH and SO_3_H groups after sonication. This phenomenon could correspond to the hydrolysis of anhydride and lactone moieties, which are frequently found in GO materials [50,51,52]. Additionally, the hydrolysis could be autocatalyzed by the surface acidic groups, which were present in the samples before the process occurred [53,54]. This process was observed particularly for the GO1 and GO2 samples, which contained sulfo (-SO_3_H) groups (pK_a_ range for aromatic sulfonic acids spans from −6.2 to −1.3 [55]). This group is indeed a stronger Brønsted–Lowry acid than the corresponding carboxylic group (-COOH) (pK_a_ range for aromatic carboxylic acids spans from 2.2 to 4.6 [56]). The hydrolysis was further enhanced by breaking up aggregates of the micrometer-size GO flakes. Hence, the diffusion-driven nucleophilic attack of water molecules onto protonated carbonyl-based (C=O) groups, i.e., anhydrides and lactones, was accelerated. The concentration of the other oxygen group determinable by Boehm titration, i.e., hydroxyl group, was found to be negligible. On the other hand, the concentration of sulfo groups in GO1 and GO2 samples after ultrasonication in water decreased to undetectable levels. These effects could be explained by the hydrolysis of the sulfo group. It is well-known that the sulfo group is susceptible to aromatic substitution by hydronium ions (H_3_O^+^) in diluted acids. The summary of this process is illustrated in Figure 17 with the final characteristics (size distribution, pH and electrical conductivity) representing an interplay between adsorption-driven GO nanosizing [31], hydrolysis of acidic precursor functional groups and desulfonation.

An important phenomenon observed after the administration of nanoparticles to blood is an interaction with plasma proteins often described as a formation of “protein corona” [18,21,57]. (Figure 16) Albumin is one of the prominent components of the mammalian blood plasma (55–69% of plasma proteins) and significantly contributes to the protein corona. Bovine serum albumin (BSA), which is frequently used in experimental studies, is composed of 583 amino acids and has 76% sequence identities with the human serum albumin. It is reported to have a high structural stability that can be recovered with pH change due to Cys disulfide bridges that protect its structure [58]. Having its isoelectric point at pH 4.6, albumin is negatively charged at a pH between 5 and 9 [59]. Although its total particle charge at pH 7 is negative (−17) [60], it expresses positively charged lysine residues (Lys537, Lys535) with an isoelectric point [59] at pH 11 that can penetrate the water layer and create a hydrogen bond with oxygen at the negatively charged surface of surrounding particles in the aqueous dispersion. Some other residues were proposed to create stabilizing contacts between BSA and the negatively charged, oxygen-rich surface itself (negative Glu494, Glu541, neutral Thr539; Thr495, Gln542 and Thr580) [60]. At a pH below 4.7, BSA was reported to unfold and present more basic amino acid residues that could modify the particle charge and also charge density [61]. Upon contact with GO, BSA fluorescence is quenched proportionally to GO concentrations and this observation depends on the GO particle size, for which fluorescence quenching was lower with smaller GO particles [62]. The alpha-helix content of BSA decreases to 59.45%, 46.60% and 18.47% upon the formation of a protein complex with GO (GO particle mean size: 955, 475 and 285 nm, respectively) [62]. In our study, for GO-BSA complexes in the aqueous dispersion, a decrease in Z-average was noted (significant for GO1 and GO2), as well as an increase in the dominant particle fraction percentage in each type of GO, and this behavior was significant for GO3 and GO4 in single-results statistical analysis. Moreover, a decrease in PDI (significant for GO1, GO2, GO3) and a lower conductivity in all types of GO-BSA dispersions were found in our study. The presence of only two particle populations in all types of GO-BSA dispersions was an important finding at this stage. The maximal particle size observed in all GO-BSA dispersions was 4000–5000 nm, which is still too large for safe tissue perfusion, but the populations of the particles of that size were only between 3.8 and 5.4% of all the particles in the dispersion (see Table 1).

The stability of the dispersion can generally be improved at a higher pH and at low concentrations of bivalent cations [44], although the proposed mechanism for cation-induced GO aggregation is different from that caused by low pH aggregation (edge-to-edge versus face-to-face interaction, respectively) [44]. In the filtering procedure elaborated to increase the GO particle retention in porous media, increased concentrations of sodium (50 mM), calcium (1 mM), magnesium (1.75 mM) and aluminum (0.03 to 0.05 mM) cations were used for an intentional GO aggregation [63]. The critical cation concentration (CCC) for inducing GO aggregation was measured by Yang [8], and, in his study, it was highly dependent on the cation valency being equal to 20 mmol/L for K^+^, 30 mmol/L for Na^+^, 0.4 mmol/L for Ca^2+^, 0.7 mmol/L for Mg^2+^ and 0.045 mmol/L for trivalent Cr^3+^, where Cr^3+^ was the most destabilizing cation used. In this study, the tested GO colloid was stable at a pH between 4 and 12. As isotonic solutions contain sodium ions at higher concentrations (135–145 mmol/L of Na^+^), the stabilization of GO in blood or tissues can still be a challenge. In our study, we observed not only a 1-hour stability of isotonic GO-BSA-KH dispersions but also a further decrease in the GO particle size, which was significant in the single-results analysis of all types of GOs. Importantly, a very high monodispersity of the final dispersions was observed. Indeed, single-particle fractions in GO1 and GO4 and a low percentage of second-particle fractions in GO3 (0.4%) and GO2 (7.5%) were found together with low PDIs in all tested GOs. The largest particles were found in the final GO3 dispersions, which presented a granular particle shape in TEM observation (1839 nm, 7.5% in particle size distribution analysis and 440.2 nm in the cumulate method). The maximal particle sizes observed in the final GO-BSA-KH dispersion (distribution analysis) of laminar-shape GOs were: 193.6 nm (GO1); 253.9 (GO2); 234 nm (GO4) and 421.1 nm; 382.6 nm; 490.1 nm in cumulate method analysis, respectively. All the laminar-shape GO forms responded well to the proposed stabilization procedure without differences between high- and low-oxygen-content particles (GO1, GO2, GO4). The observations presented here on the stabilization of various types of graphene oxide particles with regard to the particle number of layers, particle shape and particle oxygen content in isotonic solution/dispersion can widen the possibilities of the safe application of graphene oxides in organ perfusion ex vivo and as a blood-transported drug in vivo. 

## 5. Conclusions

Bovine serum albumin can be used as a dispersion stabilizer in the presence of calcium, magnesium and sodium in isotonic, precipitating concentrations of these ions. Since BSA is a major component of blood plasma proteins, the possibility of the direct stabilization of GO nanoparticles in blood is a promising concept and should be further tested. BSA in our study protocol was the effective stabilizer for GO nanoparticles with a variable number of atom layers as well as variable oxygen and nitrogen content.

## Figures and Tables

**Table 1:** The averaged particle sizes of GO1, GO2, GO3 and GO4 of GO aqueous dispersions in the consecutive preparation stages. Samples (10) were measured using a DLS method (measurement by intensity) three times each (at 37 °C). The number of measurements is shown in brackets, if different from 30. The percentages represent three main particle populations detected in the averaged samples. In GO4, additional samples were tested because of a different pattern of the changes observed. If the percentage is below 100% for all three particle fractions, it means that more than three particle fractions were detected. Abbreviations: Z-Ave—Z-average: the average particle size calculated with the cumulate method for sample population (expressed in nm as the mean value for all measurements for given type of GO and stage of the preparation procedure); PDI: polydispersity index expressed as the mean value for given stage and type of the GO; Averaged Int1–3: the size (d.nm—diameter in nanometers) of three biggest particle subpopulations of GO in given stage and type of GO; Area Int1–3%: the percentage of the whole measured sample population calculated for three main subpopulations (calculated from particle size distribution of pooled population of all samples representing given type of the GO at given stage of preparation procedure).

Sample Name, Stage and Number (Averaged Data)	Z-Ave (d.nm)	PDI	Averaged Int 1 (d.nm)	Averaged Int 2 (d.nm)	Averaged Int 3 (d.nm)	Area Int 1 (%)	Area Int 2 (%)	Area Int 3 (%)
GO Stock								
GO1 pu	3557.0	0.916	740.1	185.1	97.6	95.0	3.0	1.3
GO2 pu	4363.0	0.668	1481.0	188.3	5119.0	33.7	27.3	21.1
GO3 pu	1077.0	0.717	1049.0	4642.0	267.2	60.8	20.5	18.7
GO4 pu	3386.0	0.844	657.3	360.4	266.9	56.7	18.7	12.4
GO Diluted								
GO1 140 µg/mL	1344.0	0.412	913.9	391.4	4526.0	63.1	21.8	9.0
GO2 140 µg/mL	1759.0	0.612	830.0	2066.0	5207.0	73.6	23.0	3.1
GO3 140 µg/mL	768.8	0.559	1163.0	265.2	4906.0	66.5	25.7	7.9
GO4 140 µg/mL (42)	26300	0.758	2347.0	1734.0	4557.0	21.7	20.7	20.0
GO Sonicated								
GO1 140 µg/mL 6 × 50 W	628.0	0.518	382.9	92.8	4417.0	92.1	5.2	2.6
GO2 140 µg/mL 6 × 50 W	654.2	0.552	197.3	508.8	54.1	79.9	19.9	0.2
GO3 140 µg/mL 6 × 50 W	532.1	0.472	767.9	4419.0	0	89.7	10.3	0
GO4 140 µg/mL 6 × 50 W (42)	536.1	0.489	751.1	4628.0	0	93.8	6.2	0
GO With BSA								
GO1 50 µg/mL BSA 400 mg/L	357.9	0.38	285.7	3981.0	0	96.2	3.8	0
GO2 50 µg/mL BSA 400 mg/L	408.2	0.418	408.6	4405.0	0	95.8	4.2	0
GO3 50 µg/mL BSA 400 mg/L	509.7	0.391	716.7	4657.0	0	94.6	5.4	0
GO4 50 µg/mL BSA 400 mg/L	441.8	0.448	486.4	4486.0	0	96.0	4.0	0
GO with BSA and Krebs–Henseleit								
GO1 10 µg/mL BSA 80 mg/L KH	421.1	0.385	193.6	0	0	100	0	0
GO2 10 µg/mL BSA 80 mg/L KH	382.6	0.363	253.9	66.9	0	99.6	0.4	0
GO3 10 µg/mL BSA 80 mg/L KH	440.2	0.406	294.4	1839.0	0	92.5	7.5	0
GO4 10 µg/mL BSA 80 mg/L KH (42)	490.1	0.435	234.0	0	0	100	0	0

**Table 2:** Analysis of the functional groups in the GO samples before and after sonication.

GO Functional Groups by Titration	COOH mmol/g	SO3H mmol/g	NH2 mmol/g	Phenol OH mmol/g
GO1				
before sonication	1.13	1.17	nt *	nt
post-sonication 6 × 50 W 1	3.5	0.1	nt	<0.1
post-sonication 6 × 50W 2	4	0.15	nt	<0.1
GO2				
before sonication	1.35	1.57	nt	nt
post-sonication 6 × 50W 1	3.7	0.1	nt	<0.1
post-sonication 6 × 50W 2	3.7	0.1	nt	<0.1
GO3				
before sonication	2.52	nd	nt	nt
post-sonication 6 × 50W 1	4.5	0.2	nt	nt
post-sonication 6 × 50W 2	4.3	0.3	nt	nt
GO4				
before sonication	2.49	nd	nt	nt
post-sonication 6 × 50W 1	4	0.1	1.2	nt
post-sonication 6 × 50W 2	3.9	0.2	1	nt

* not tested.

## Data Availability

The data presented in this study are available on request from the corresponding author. The data are not publicly available due to ongoing further analysis.
